# Impact of a Prospective Audit and Feedback Antimicrobial Stewardship Program at a Veterans Affairs Medical Center: A Six-Point Assessment

**DOI:** 10.1371/journal.pone.0150795

**Published:** 2016-03-15

**Authors:** Haley J. Morrill, Aisling R. Caffrey, Melissa M. Gaitanis, Kerry L. LaPlante

**Affiliations:** 1 Veterans Affairs Medical Center, Infectious Diseases Research Program, Providence, Rhode Island, United States of America; 2 University of Rhode Island, Department of Pharmacy Practice, College of Pharmacy, Kingston, Rhode Island, United States of America; 3 Veterans Affairs Medical Center, Center of Innovation in Long Term Services and Supports, Providence, Rhode Island, United States of America; 4 Warren Alpert Medical School of Brown University, Division of Infectious Diseases, Providence, Rhode Island, United States of America; University of Calgary, CANADA

## Abstract

**Background:**

Prospective audit and feedback is a core antimicrobial stewardship program (ASP) strategy; however its impact is difficult to measure.

**Methods:**

Our quasi-experimental study measured the effect of an ASP on clinical outcomes, antimicrobial use, resistance, costs, patient safety (adverse drug events [ADE] and *Clostridium difficile* infection [CDI]), and process metrics pre- (9/10–10/11) and post-ASP (9/12–10/13) using propensity adjusted and matched Cox proportional-hazards regression models and interrupted time series (ITS) methods.

**Results:**

Among our 2,696 patients, median length of stay was 1 day shorter post-ASP (5, interquartile range [IQR] 3–8 vs. 4, IQR 2–7 days, p<0.001). Mortality was similar in both periods. Mean broad-spectrum (-11.3%), fluoroquinolone (-27.0%), and anti-pseudomonal (-15.6%) use decreased significantly (p<0.05). ITS analyses demonstrated a significant increase in monthly carbapenem use post-ASP (trend: +1.5 days of therapy/1,000 patient days [1000PD] per month; 95% CI 0.1–3.0). Total antimicrobial costs decreased 14%. Resistance rates did not change in the one-year post-ASP period. Mean CDI rates/10,000PD were low pre- and post-ASP (14.2 ± 10.4 vs. 13.8 ± 10.0, p = 0.94). Fewer patients experienced ADEs post-ASP (6.0% vs. 4.4%, p = 0.06).

**Conclusions:**

Prospective audit and feedback has the potential to improve antimicrobial use and outcomes, and contain bacterial resistance. Our program demonstrated a trend towards decreased length of stay, broad-spectrum antimicrobial use, antimicrobial costs, and adverse events.

## Introduction

Antimicrobial resistance is one of the greatest public health threats worldwide.[1] In the United States (US), the Obama Administration recently identified antimicrobial resistance as a national security issue.[2] Infections with antimicrobial-resistant bacteria and *Clostridium difficile* lead to increased morbidity, mortality, longer hospital stays, and dramatically increased healthcare costs.[3–5] The Centers for Disease Control and Prevention estimated that in 2013, antimicrobial-resistant organisms caused two million infections and 23,000 deaths in the US, with an additional 14,000 deaths due to *C*. *difficile* infection (CDI).[1] In the US, resistant infections are responsible for $20–35 billion in excess healthcare costs each year.[1]

The driving forces that select for antimicrobial-resistant bacteria and promote CDI are antimicrobial use and suboptimal infection control practices. While some cases of CDI are not associated with prior antibiotic use and many other risk factors for CDI exist, including advanced age and protein pump inhibitor use, antibiotic use remains the most important risk factor for the development of CDI.[6] Given that over 50% of antimicrobial use in hospitals may be inappropriate, antimicrobial stewardship interventions (coordinated strategies to improve antimicrobial use) are critically important.[7] The 2007 Infectious Diseases Society of America and Society for Healthcare Epidemiology of America guidelines for developing an antimicrobial stewardship program (ASP) have recognized prospective audit of antimicrobial use and feedback back to the prescriber and formulary restriction as two core strategies that provide the foundation of an ASP.[7] Literature supports the effectiveness of prospective audit and feedback, however measuring the impact of these programs has been difficult.[8–17] Therefore, the purpose of this study was to conduct a broad evaluation of a prospective audit and feedback ASP on the following six measures: 1) clinical outcomes, 2) antimicrobial utilization, 3) costs, 4) resistance, 5) patient safety (adverse drug events [ADE] and CDI), and 6) process metrics. To our knowledge, our study is one of the first published studies to provide a comprehensive six-point assessment on the impact of an ASP.

## Methods

We conducted a single-center quasi-experimental study. Study metrics were compared pre-(9/2010-10/2011) and post-ASP (9/2012-10/2013). The study protocol was approved by the Institutional Review Board and the Research (IRB) and Development Committee of the Providence Veterans Affairs Medical Center (PVAMC). The PVAMC IRB specifically waived the need for written informed consent for this retrospective study as it met the requirements of 38 CFR 16.116(d).

### Intervention

The PVAMC is a Veterans Affairs (VA) teaching hospital licensed for 119 beds. In September 2012, the PVAMC invested in and implemented a formal ASP. Prior to formal introduction, the program was pilot tested for ~18 weeks between 10/2011-4/2012. After that time the PVAMC funded a new ID fellowship position for pharmacists focusing in ASP. The new ID pharmacist fellow began in July 2012 and spent the next two months writing the policy and getting it approved by hospital administration. The ID pharmacist fellow began prospective audit and feedback in September 2012. A second new ID pharmacist fellow joined the team in July 2013. The core members of the program included the co-directors (a board certified infectious diseases [ID] attending physician and a clinical pharmacist with formal ID fellowship training), two other board certified ID attending physicians, two ID pharmacist fellows, and when on rotation, ID physician fellows (~6 months), PGY-1 pharmacy practice residents and APPE students (~9 months). Infection control practitioners, microbiology laboratory personnel, and an epidemiologist supported the core team. The main strategy implemented by the ASP was prospective audit and feedback. Since formal introduction, core team members have provided prospective audit and feedback for every patient admitted with active antimicrobial orders (Monday-Friday).

The on-service ID pharmacist fellow manually reviewed a list of all active antimicrobial orders daily. The list was generated in the morning and all active orders were reviewed with no restrictions for how long the patient was on the antibiotic before review. Each antimicrobial order was reviewed for appropriateness. Appropriateness was determined by the ID pharmacist fellow, who reviewed each order to make sure the correct drug, dose, duration, and/or route were used. The ID pharmacist fellow also ensured there was an indication for the antimicrobial order. No single definition for appropriateness was instituted, however the ID pharmacist fellow utilized institutional guidelines (PVAMC Antimicrobial Treatment Guidebook) professional society guidelines, expert opinion of the ASP core members (pre-rounding with an ID physician and/or the senior clinical pharmacist), and local and regional resistance patterns to determine appropriateness.[7]

**Fig 1:**
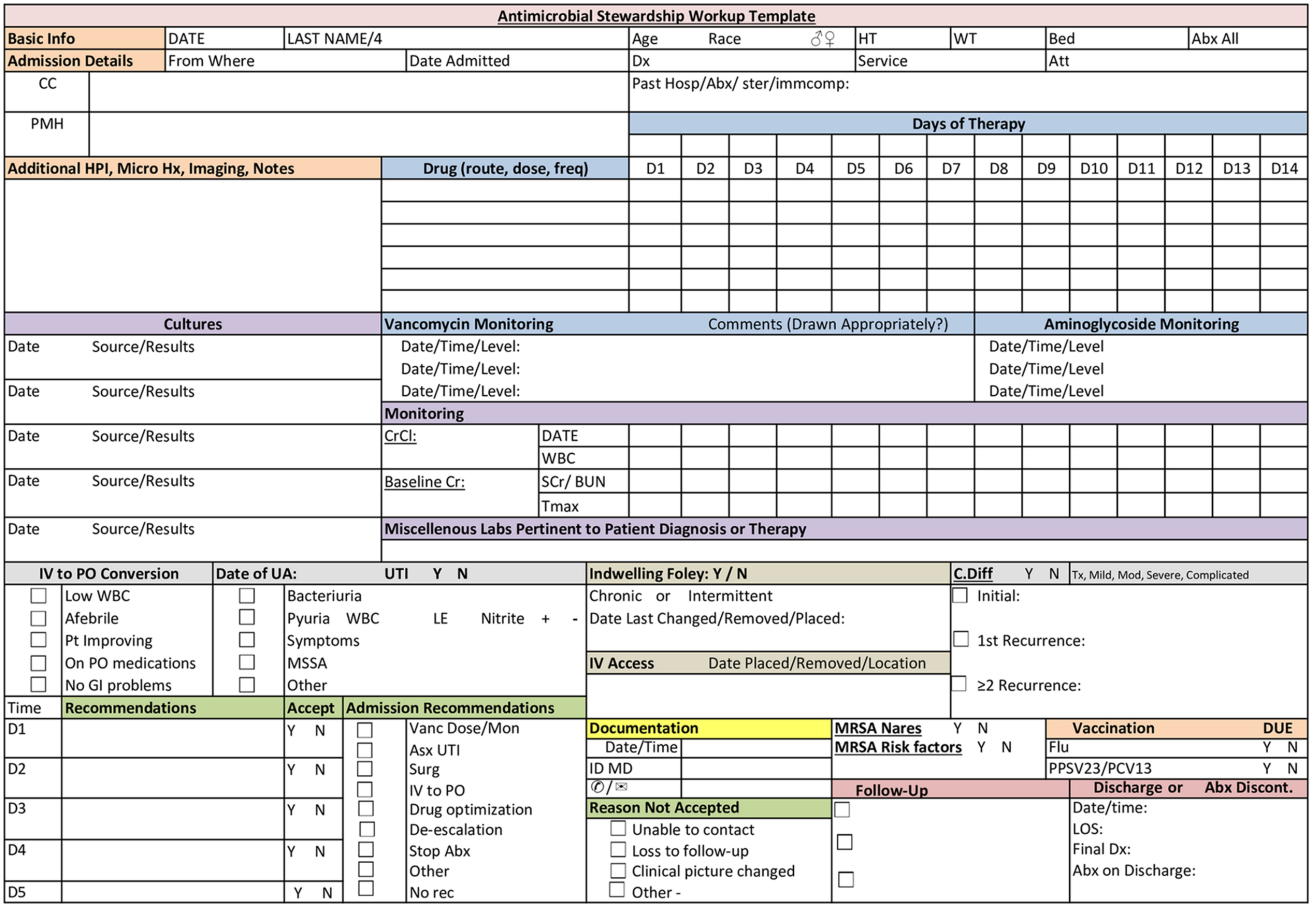
Antimicrobial Stewardship Patient Workup Template.

The PVAMC has published an annually updated Antimicrobial Treatment Guidebook since 2004, which contains empiric treatment guidelines, dosing recommendations, infection control policies, and an antibiogram of antimicrobial resistance rates. Additionally, a pre-designed decision-support template was used to collect and organize pertinent clinical data for ASP interventions (Fig 1). Other antimicrobial stewardship principles such as intravenous (IV) to oral (PO) conversion, de-escalation of empiric therapy based on culture results, and antimicrobial optimization were used to make recommendations to improve “appropriateness”.[7] Antimicrobial optimization involved recommendations to improve the drug, dose, or duration of the antimicrobial based on patient characteristics, causative organism, site/type of infection, and pharmacokinetic/pharmacodynamics characteristics. Potential interventions were then relayed to the on-service ID physician and/or the senior clinical pharmacist. These “ASP rounds” were conducted daily and generally ranged from 15–60 minutes.

After discussing patients and interventions, verbal communication (telephone and in-person) and/or written notes in the electronic medical record (EMR) were used to relay interventions to the provider. The mode of communication (verbal or written by physician, pharmacist, or pharmacy resident/student) depended on the type of intervention that was needed. The specific intervention also dictated who made the intervention (physician, pharmacist, or pharmacy resident/student). For example, for a simple IV to PO antimicrobial conversion (e.g. IV to PO ciprofloxacin), a pharmacy student or resident may have written a draft note. However, discontinuation of an antimicrobial in a complex patient may have necessitated a phone call to the primary team by the on-service ID physician. To alert the provider (usually the medical resident) of the note, they were added as co-signers. The ID pharmacy fellow fully reviewed and signed-off on all notes written by residents and students before they were incorporated into the EMR. Additionally, the ID pharmacy fellow alerted the on-service ID physician to all written notes for review and co-signature.

### Process Metrics

During the post-study period, the on-service ID pharmacy fellow documented all patients that were reviewed by the ASP in an excel database. Variables collected included admission date, treating specialty, antimicrobial indication, time-spent, and whether an intervention was made. If an intervention was made, the pharmacy fellow documented the type of intervention made, the stewardship team member who made the intervention, intervention acceptance or non-acceptance, and reasons for non-acceptance. Acceptance or non-acceptance was qualified as a dichotomous variable for each recommendation made. Interventions were categorized as follows: vancomycin dosing or therapeutic drug monitoring, antimicrobial discontinuation, IV to PO conversion, de-escalation, antimicrobial optimization (i.e. change to optimize the antimicrobial drug, dose, or duration), antimicrobial discontinuation, or other.

### Clinical Outcomes

Clinical outcomes were compared between patients pre- and post-ASP. We identified all hospital inpatients with antimicrobials administered during the pre- and post-ASP periods.[18] Patients with a long-term stay (≥ 90 days) were excluded. Inpatient antimicrobial administrations were captured using patient barcode medication administration (BCMA) data.

Outcomes included time to hospital discharge (length of stay [LOS]), 7-, 14-, and 30-day all-cause mortality, inpatient all-cause mortality, and 30-day readmission. The index date for hospital discharge, 7-, 14- and 30-day mortality, and inpatient mortality was the date of antimicrobial initiation and for 30-day readmission was the date of hospital discharge. We calculated the time from the index date to the date of event for each outcome. Patients were censored on their date of death.

We determined demographics, comorbid conditions, and health-care exposures from the national VA standardized databases which contain ICD-9 diagnostic and procedure codes, vital status, microbiology results, barcode medication administration, and laboratory results.

### Antimicrobial Utilization

Antimicrobial utilization was compared pre- and post-ASP. The antimicrobial utilization metric used was days of therapy per 1,000 patient days (DOT/1000PD) based on inpatient medication administration data.[19, 20] We assessed overall antimicrobial use, as well as specific categories of use by route, agent, class, and spectrum.[21]

### Antimicrobial Costs

Antimicrobial costs were estimated using the Average Wholesale Price. The cost metric used was cost per 1,000 patient-days. Overall costs and costs for specific antimicrobial categories described above were compared pre- and post-ASP.

### Antimicrobial Resistance

Antimicrobial resistance was assessed using PVAMC culture and susceptibility data (antibiogram). Antimicrobial resistance for several important organism-antimicrobial combinations tested at the PVAMC were compared pre- and post-ASP.[1] The organisms assessed included *Enterococcus faecalis*, *Enterococcus faecium*, methicillin-susceptible *Staphylococcus aureus* (MSSA), MRSA, *Klebsiella pneumoniae*, *Acinetobacter baumannii*, *Pseudomonas aeruginosa*, and *Escherichia coli*.

### Patient Safety

Monthly episodes of CDI per 10,000 patient-days were compared pre- and post-ASP. CDI episodes were obtained from VA Inpatient Evaluation Center (IPEC) data.[22, 23] Rates of ADEs among hospital inpatients with antimicrobial administrations were compared pre- and post-ASP. ADEs were identified using ICD-9 codes for adverse effects of drugs.

### Statistical Analysis

All analyses were performed using SAS (SAS Institute Inc., Cary, NC, Version 9.2).

#### Process metrics

We used descriptive statistics, including means and percentages, to summarize the data.

#### Clinical outcomes

Baseline differences between patients in the pre- and post-ASP periods were assessed using Fisher’s exact or *χ*^*2*^ tests (categorical data), and a t-test or Wilcoxon Rank Sum test (continuous data), as appropriate. Propensity score adjustment and matching was implemented to balance differences between patients in the pre and post-ASP periods.[24, 25] Propensity scores were developed from an unconditional logistic regression model (manual backward elimination). Hazards ratios comparing clinical outcomes in post-ASP patients to pre-ASP patients were calculated from Cox proportional-hazards regression models.

#### Antimicrobial utilization

T-tests were used to compare mean DOT/1000PD pre- and post-ASP. We utilized interrupted time series (ITS) methods to assess the impact of ASP on monthly antimicrobial utilization. Segmented linear regression models were used because they can tolerate fewer time points than autoregressive integrated moving average models.[26, 27] We tested for autocorrelation using the Durbin-Watson statistic, and for seasonality/stationarity using the Dickey-Fuller unit root test.[27, 28] Estimates for regression coefficients corresponding to the effect sizes of a change in level and a change in trend for post- to pre-ASP were obtained. A change in level was defined as the difference between the observed level immediately post-ASP and the predicted level by the pre-ASP trend. A change in trend was defined as the difference between the pre and post-ASP slopes.

#### Antimicrobial costs

T-tests were used to compare mean costs/1000PD pre- and post-ASP. Segmented linear regression models were utilized to model temporal trends in monthly antimicrobial costs.

#### Antimicrobial resistance

We used Fisher’s exact or *χ*^*2*^ tests, as appropriate, to compare the number of resistant and susceptible isolates for select organism-antimicrobial combinations pre- and post-ASP.

#### Patient safety

A t-test was used to compare mean CDI rate/10,000PD and the *χ*^*2*^ test was used to compare ADEs pre- and post-ASP. Segmented linear regression models were utilized to model temporal trends in monthly CDI rates.

## Results

### Process Metrics

**Fig 2:**
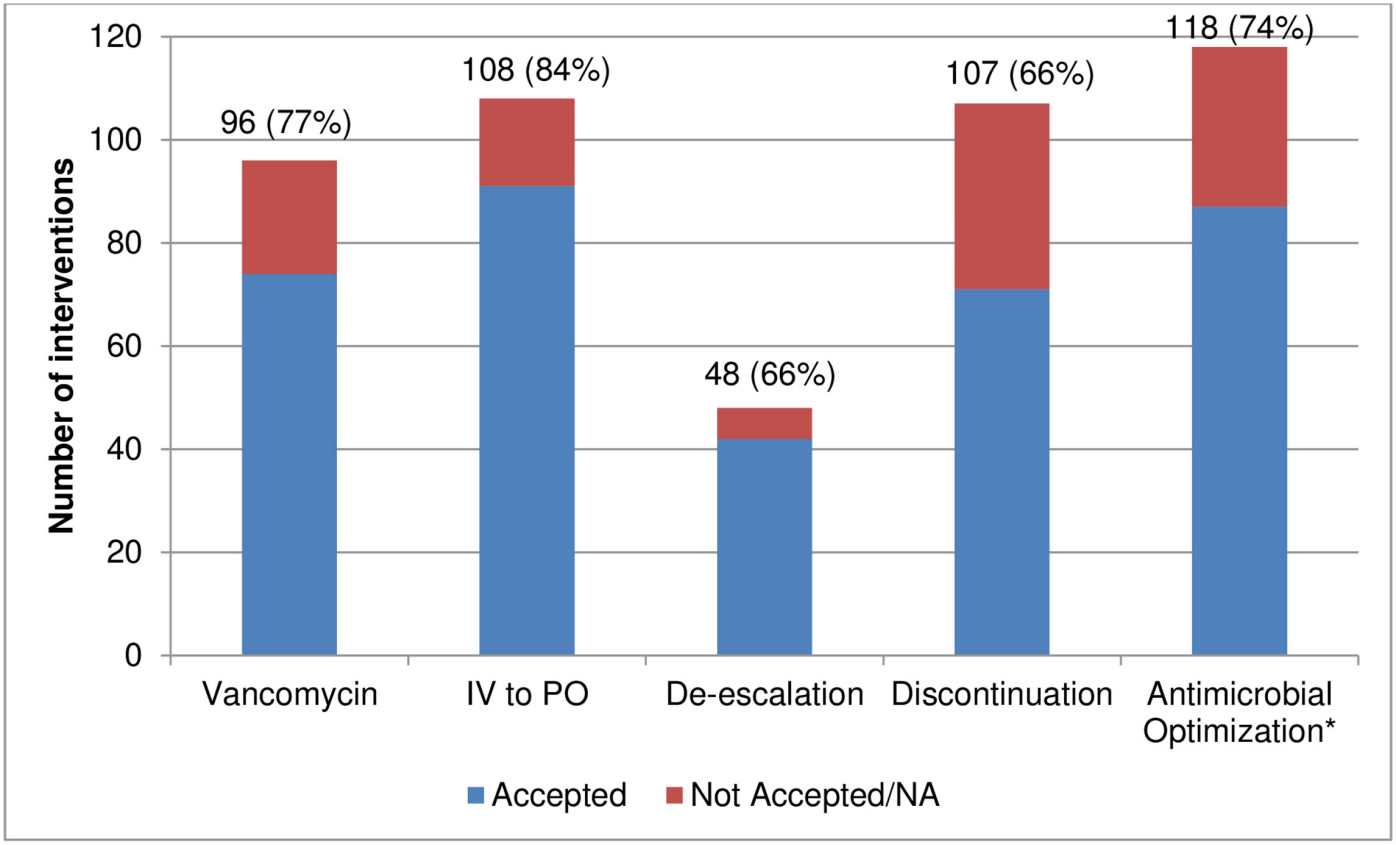
Antimicrobial Stewardship Interventions and Acceptance Rates. Data expressed as number of interventions (% accepted). IV = Intravenous; PO = Oral; NA = Intervention no longer appliable, for example patient discharged home, or antibiotic of interest was switched or discontinued. * = Antimicrobial optimization includes any recommendation to improve the drug, dose, or duration of an antimicrobial.

During the post-ASP period, we reviewed 1,049 patient charts. Interventions were made in 36.7% of patients reviewed. The most common interventions made were antimicrobial optimizations, IV to PO conversions, and discontinuations (Fig 2). Among the patients with an intervention, interventions were most often (88.3%) made through a written note in the patients’ EMR. The on-service pharmacy fellow made the intervention in almost half of the patients who needed an intervention (47.8%). Overall, 522 interventions were made with an overall acceptance rate of 77.2%. The most common reasons for non-acceptance, were that the primary team never viewed the recommendation (29%) or that the antimicrobial was changed/discontinued (14%) and therefore the recommendation was no longer applicable.

### Clinical Outcomes

We identified 2,696 patients treated with antimicrobials in the pre- (49.0%, n = 1,321) and post-ASP (51.0%, n = 1,375) periods. The median patient age was ~70 years in both groups (Table 1). The median Charlson (2 vs. 4) and Elixhauser (3 vs. 5) scores were higher for patients post-ASP (p<0.001). History, in the year prior to the antibiotic-related admission, of diabetes, congestive heart failure, myocardial infarction, chronic respiratory disease, and chronic renal disease were more common among post-ASP patients. Diagnoses of cellulitis, osteomyelitis, and influenza during the current admission were more common for patients post-ASP (Table 1). More post-ASP patients were hospitalized in the 90 days prior to admission than pre-ASP (Table 2). Despite these differences between pre- and post-ASP patients, we were able to balance significantly different baseline characteristics using propensity scores.

**Table 1:** Demographics and Comorbid Conditions by Period.

Demographic characteristics	Pre-Antimicrobial Stewardship Period (n = 1,321)	Post-Antimicrobial Stewardship Period (n = 1,375)
Age (years)	71.0 (62.0–82.0)	70.0 (62.0–82.0)
Male Gender	1,289 (97.6)	1,324 (96.3)
White Race	1,208 (91.4)	1,280 (93.1)
Ethnicity		
Non-Hispanic	1,293 (97.9)	1,338 (97.3)
Hispanic	7 (0.5)	19 (1.4)
Unknown	21 (1.6)	18 (1.3)
Marital Status*		
Married	484 (36.6)	497 (36.1)
Divorced / Separated	401 (30.4)	454 (33.0)
Widowed	224 (17.0)	261 (19.0)
Single/ Never Married	206 (15.6)	154 (11.2)
Unknown	6 (0.5)	9 (0.7)
Body Mass Index*		
<18.5	35 (2.6)	15 (1.1)
18.5–24.9	560 (42.4)	622 (45.2)
25.0–29.9	257 (19.5)	246 (17.9)
30+	435 (32.9)	475 (34.5)
Unknown	34 (2.6)	17 (1.2)
Charlson Score*	2.0 (1.0–4.0)	4.0 (2.0–6.0)
Elixhauser Score*	3.0 (1.0–5.0)	5.0 (3.0–7.0)
Medical History within One Year Prior to the Antibiotic-Related Admission		
Alcohol Abuse*	216 (16.4)	295 (21.5)
Amputation*	46 (3.5)	78 (5.7)
Any Cancer	310 (23.5)	361 (26.3)
Burns*	< 5	12 (0.9)
Cardiac Arrhythmia*	386 (29.2)	461 (33.5)
Chronic Renal Disease*	240 (18.2)	274 (19.9)
Chronic Respiratory Disease*	528 (40.0)	662 (48.1)
Chronic Ulcer*	145 (11.0)	232 (16.9)
Complication of Implant or Graft	71 (5.4)	89 (6.5)
Congestive Heart Failure	303 (22.9)	346 (25.2)
Coronary Heart Disease*	430 (32.6)	497 (36.1)
Depression*	460 (34.8)	657 (47.8)
Diabetes*	461 (34.9)	539 (39.2)
Drug Abuse*	133 (10.1)	168 (12.2)
Fluid and Electrolyte Disorders*	357 (27.0)	551 (40.1)
Gangrene*	16 (1.2)	41 (3.0)
Hypertension*	774 (58.6)	982 (71.4)
Hypothyroidism*	83 (6.3)	125 (9.1)
Immunity Disorder	< 5	< 5
Metastatic Cancer	51 (3.9)	49 (3.6)
Mild Liver Disease*	85 (6.4)	144 (10.5)
Moderate/Severe Liver Disease*	25 (1.9)	47 (3.4)
Myocardial Infarction*	107 (8.1)	150 (10.9)
Neutropenia	12 (0.9)	14 (1.0)
Paraplegia and Hemiplegia	44 (3.3)	71 (5.2)
Peptic Ulcer Disease*	34 (2.6)	68 (4.9)
Peripheral Vascular disease*	181 (13.7)	264 (19.2)
Psychoses*	106 (8.0)	172 (12.5)
Pulmonary Circulation Disorders*	57 (4.3)	104 (7.6)
Rheumatoid Arthritis	26 (2.0)	27 (2.0)
Surgery/Medical Complication*	68 (5.1)	183 (13.3)
Tobacco Abuse*	251 (19.0)	371 (27.0)
Infection Diagnosis (ICD-9) During Antibiotic-Related Admission^a^		
Bacteremia	49 (3.7)	49 (3.6)
Cellulitis or Abscess*	157 (11.9)	199 (14.5)
Endocarditis	< 5	6 (0.4)
Influenza*	< 5	49 (3.6)
Osteomyelitis*	21 (1.6)	41 (3.0)
Pneumonia	284 (21.5)	304 (22.1)
Skin/Subcutaneous Infection	265 (20.1)	318 (23.1)
Urinary Tract Infections	325 (24.6)	326 (23.7)
Culture/Laboratory Confirmed Infections During Antibiotic-Related Admission		
Bacteremia^b^	59 (4.5)	46 (3.3)
*Clostridium difficile* Infection^c^	73 (5.5)	85 (6.2)
Influenza^c^*	< 5	32 (2.3)
Pneumonia^c^	45 (3.4)	49 (3.6)
Skin/Subcutaneous Tissue Infection^c^	50 (3.8)	72 (5.2)
Urinary Tract Infection^c^	170 (12.9)	168 (12.2)
Positive Culture During Antibiotic-Related Admission^d^		
*Enterococcus faecalis*	24 (1.8)	30 (2.2)
VRE*	8 (0.6)	< 5
MSSA	42 (3.2)	39 (2.8)
MRSA	42 (3.2)	45 (3.3)
*Streptococcus* species*	5 (0.4)	30 (2.2)
*Escherichia coli*	61 (4.6)	48 (3.5)
*Klebsiella pneumoniae*	47 (3.6)	37 (2.7)
*Pseudomonas aeruginosa*	55 (4.2)	38 (2.8)
Fungal species*	< 5	50 (3.6)
Previous Infection Diagnosis (ICD-9) within One Year Prior to the Antibiotic-Related Admission^a^		
Bacteremia	41 (3.1)	59 (4.3)
Cellulitis or Abscess*	168 (12.7)	282 (20.5)
Gram-negative*	37 (2.8)	70 (5.1)
Influenza*	5 (0.4)	32 (2.3)
Osteomyelitis*	24 (1.8)	77 (5.6)
Pneumonia*	197 (14.9)	327 (23.8)
Pseudomonas*	17 (1.3)	52 (3.8)
Skin/ Subcutaneous Tissue Infections*	264 (20.0)	416 (30.3)
Staphylococcus aureus*	19 (1.4)	45 (3.3)
MRSA	39 (3.0)	59 (4.3)
*Streptococcus* species*	27 (2.0)	63 (4.6)
Surgical Site Infection*	11 (0.8)	48 (3.5)
Urinary Tract Infections*	238 (18.0)	362 (26.3)
Previous Culture/Laboratory Confirmed Infections within One Year Prior to the Antibiotic-Related Admission		
Bacteremia^b^	74 (5.6)	66 (4.8)
Bone and Joint*^c^	14 (1.1)	33 (2.4)
*Clostridium difficile* Infection^c^	77 (5.8)	96 (7.0)
Influenza*^c^	< 5	15 (1.1)
Pneumonia*^c^	33 (2.5)	61 (4.4)
Skin/Subcutaneous Tissue Infection*^c^	65 (4.9)	126 (9.2)
Urinary Tract Infections*^c^	178 (13.5)	286 (20.8)
Previous Positive Culture within One Year Prior to the Antibiotic-Related Admission^d^		
Enterococcus faecalis*	37 (2.8)	75 (5.5)
VRE*	16 (1.2)	< 5
MSSA	72 (5.5)	75 (5.5)
MRSA	86 (6.5)	80 (5.8)
*Streptococcus* species*	< 5	33 (2.4)
*Escherichia coli*	88 (6.7)	110 (8.0)
*Klebsiella pneumoniae*	95 (7.2)	95 (6.9)
*Pseudomonas aeruginosa*	73 (5.5)	81 (5.9)
Fungal species*	< 5	48 (3.5)

Data are mean ± standard deviation, median (interquartile range), or number (%) of patients.

Differences assessed by Fisher’s exact or *χ*^*2*^ test (categorical data), t-test or Wilcoxon Rank Sum test (continuous data) as appropriate.

MSSA = methicillin-sensitive *Staphylococcus aureus*; MRSA = methicillin-resistant *Staphylococcus aureus*; VRE = vancomycin-resistant *Enterococcus*.

* = p<0.05.

^a^ = Infection defined by presence of ICD-9 code.

^b^ = Bacteremia defined by positive blood culture from any organism excluding coagulase-negative *Staphylococcus* species.

^c^ = Infection defined by presence of ICD-9 code and positive corresponding culture.

^d^ = Positive culture from any site.

**Table 2:** Healthcare and Antibiotic Exposures and Hospitalization-Related Characteristics by Period.

Healthcare and Antibiotic Exposures and Hospitalization-Related Characteristics	Pre-Antimicrobial Stewardship Period (n = 1,321)	Post-Antimicrobial Stewardship Period (n = 1,375)
Treatment specialty		
Intensive Care Unit	124 (9.4)	116 (8.4)
General Medicine	1013 (76.7)	1084 (78.8)
Surgery	140 (10.6)	125 (9.1)
Other	44 (3.3)	50 (3.6)
Antibiotic Exposures during the current Admission		
Piperacillin/Tazobactam	379 (28.7)	353 (25.7)
Vancomycin	383 (29.0)	393 (28.6)
3rd/ 4^th^ Generation Cephalosporins	274 (20.7)	286 (20.8)
Beta-lactam/ Beta-Lactamase Inhibitors	411 (31.1)	401 (29.2)
Fluoroquinolones*	537 (40.7)	452 (32.9)
Carbapenems	41 (3.1)	28 (2.0)
Anti-Anaerobic Antimicrobials*^a^	733 (55.5)	690 (50.2)
Anti-Atypical Antimicrobials^b^	793 (60.0)	762 (55.4)
Anti-MRSA Antimicrobials^c^	402 (30.4)	406 (29.5)
Anti-Pseudomonal Antimicrobials*^d^	636 (48.1)	569 (41.4)
Anti-Influenza Antimicrobials*^e^	7 (0.5)	68 (4.9)
Intravenous Route Antimicrobials	924 (69.9)	905 (65.8)
Digestive Route Antimicrobials^f^	937 (70.9)	1005 (73.1)
Length of Stay (days)*	5.0 (3.0–8.0)	4.0 (2.0–7.0)
Days of Therapy*	5.0 (2.0–9.0)	4.0 (2.0–8.0)
Any Surgery During the Antibiotic-Related admission	189 (14.3)	163 (11.9)
Laboratory Results during the antibiotic-related Admission		
Maximum temperature (°F)	98.4 (98.0–99.2)	98.4 (98.0–99.1)
Maximum WBC Count (cells 10^3^/mm^3^)	9.5 (7.2–12.7)	9.5 (7.2–12.8)
Previous Antibiotics, 90 days	417 (31.6)	462 (33.6)
Previous Antibiotics, 365 days*	655 (49.6)	729 (53.0)
Previous Hospitalization, 90 days*	609 (46.1)	720 (52.4)
Previous Hospitalization, 365 days*	891 (67.4)	983 (71.5)
Previous Any Surgery, 90 days	120 (9.1)	147 (10.7)
Previous Pneumococcal Vaccine, 5 years*	222 (16.8)	593 (43.1)
Previous Influenza Vaccine, 1 year*	874 (66.2)	980 (71.3)

Data are mean ± standard deviation, median (interquartile range), or number (%) of patients.

Differences assessed by Fisher’s exact or *χ*^*2*^ test (categorical data), t-test or Wilcoxon Rank Sum test (continuous data) as appropriate.

MRSA = methicillin-resistant *Staphylococcus aureus*; WBC = White Blood Cell.

* = p<0.05.

^a^ = Antimicrobials with activity against anaerobes, included tigecycline, β-lactams/ β-lactamase inhibitors, cefoxitin, cefotetan, carbapenems, clindamycin, moxifloxacin, and metronidazole.

^b^ = Antimicrobials with activity against atypical pneumonia pathogens, included tetracyclines, tigecycline, macrolides, and fluoroquinolones.

^c^ = Antimicrobials with activity against MRSA, included tigecycline, daptomycin, telavancin, vancomycin IV, quinupristin/dalfopristin, linezolid, and ceftaroline.

^d^ = Antimicrobials with activity against *Pseudomonas aeruginosa*, included ticarcillin/clavulanate, piperacillin/tazobactam, ceftazidime, cefepime, imipenem, meropenem, doripenem, amikacin, gentamicin, tobramycin, ciprofloxacin, levofloxacin, polymyxin B, colistin, and fosfomycin.

^e^ = Antimicrobials with activity against Influenza, included oseltamivir.

^f^ = Digestive route included oral and rectal antimicrobials.

The median LOS was 1 day shorter post-ASP (5 days, IQR 3–8 vs. 4, IQR 2–7; p<0.001). In unadjusted analysis, time to discharge (LOS) was significantly shorter post-ASP (Table 3; HR 1.18, 95% CI 1.09–1.27). Unadjusted 30-day readmission was significantly higher post-ASP (HR 1.24, 95% CI 1.08–1.42). However, there was no difference in the propensity adjusted and matched analyses (550 matched pairs) for time to discharge or 30-day readmission. While all-cause 7- and 14- day mortality were similar between the two periods in all analyses, 30-day mortality was greater post-ASP in propensity adjusted analyses (HR 1.42, 95% CI 1.02–1.97); however a difference was not observed in unadjusted or propensity matched analyses.

**Table 3:** Outcomes: Post-Antimicrobial Stewardship Period Compared with Pre- Antimicrobial Stewardship Period.

Outcome	No. of events/ No. of patients Post-ASP	No. of events/ No. of patients Pre-ASP	HR (95% CI)
All-cause 7-Day Mortality			
Unadjusted	33/1,373	25/1,321	1.271 (0.756–2.137)
Adjusted	33/1,373	25/1,321	1.170 (0.616–2.221)
Matched	11/550	11/550	1.000 (0.434–2.307)
All-cause 14-Day Mortality			
Unadjusted	72/1,373	52/1,321	1.339 (0.937–1.913)
Adjusted	72/1,373	52/1,321	1.419 (0.919–2.191)
Matched	29/550	21/550	1.333 (0.757–2.348)
All-cause 30-Day Mortality			
Unadjusted	118/1,373	92/1,321	1.244 (0.948–1.634)
Adjusted	118/1,373	92/1,321	1.415 (1.016–1.971)
Matched	52/550	37/550	1.389 (0.905–2.132)
All-cause Inpatient Mortality			
Unadjusted	22/1,373	35/1,321	0.721 (0.423–1.230)
Adjusted	22/1,373	35/1,321	0.601 (0.302–1.195)
Matched	6/550	13/550	0.500 (0.125–1.999)
Discharge			
Unadjusted	1,351/1,373	1,286/1,321	1.177 (1.091–1.271)
Adjusted	1,351/1,373	1,286/1,321	1.029 (0.936–1.130)
Matched	544/550	537/550	1.100 (0.938–1.290)
30-Day Readmission			
Unadjusted	448/1,373	361/1,321	1.238 (1.077–1.422)
Adjusted	448/1,373	361/1,321	1.156 (0.975–1.370)
Matched	160/550	155/550	1.081(0.856–1.365)

CI = confidence interval; HR = hazard ratio; Pre- = Pre-Antimicrobial Stewardship Period; Post- = Post-Antimicrobial Stewardship Period.

Adjusted by propensity score quintiles (reference quintile I).

Propensity score matched within 0.001 caliper.

The propensity was derived from an unconditional logistic regression model controlling for (C-statistic 0.84) antimicrobials in the previous 90 days, hospitalization in the previous 90 days, age, current complication of surgery or medical care, antimicrobials in the previous 30 days, antimicrobials in the previous 365 days, current piperacillin/tazobactam exposure, body mass index category, current adverse drug event, current alcohol abuse, current arrhythmia, current cancer, current cerebrovascular disorder, current coronary heart disease, current congestive heart failure, current coagulopathy, current chronic renal disease, current chronic respiratory disease, current tobacco use, current deficiency anemia, current human immunodeficiency virus, current history of tobacco use, current cellulitis or abscess, current bacteremia, current influenza infection, current methicillin-resistant *Staphylococcus aureus* infection, current skin/subcutaneous infection, current urinary tract infection, current pulmonary circulation disorder, current positive coagulase-negative *Staphylococcus* culture, current positive *Escherichia coli* culture, current positive *Pseudomonas aeruginosa* culture, current positive *Streptococcus* species culture, current rheumatoid arthritis, current valvular disease, current Elixhauser score, creatinine, days of antimicrobial therapy, ethnicity, current beta-lactam/ beta-lactamase inhibitor exposure, current anti-influenza drug exposure, current fluoroquinolone exposure, current macrolide exposure, current metronidazole exposure, current tetracycline class exposure, current digestive route antimicrobial exposure, current anti-atypical drug exposure, current anti-*Clostridium difficile* drug exposure, current other antimicrobial exposure, gender, previous alcohol abuse, previous burn, pervious coronary heart disease, previous chronic ulcer, previous coagulopathy, previous chronic renal disease, previous tobacco use, previous deficiency anemia, previous diabetes mellitus, previous drug abuse, previous endocarditis, previous human immunodeficiency virus, previous hypertension, previous history of tobacco use, previous cellulitis or abscess, previous bacteremia, previous Gram negative infection, previous influenza infection, previous pneumonia, previous *Pseudomonas* species infection, previous *Staphylococcus aureus* infection, previous surgical site infection, previous *Streptococcus* species infection, previous urinary tract infection, previous severe liver disease, previous obesity, previous other neurologic disorder, previous osteomyelitis, previous positive blood culture, previous positive catheter tip culture, previous positive other site culture, previous positive skin culture, previous positive *Proteus* species culture, previous positive *Streptococcus* culture, previous positive *Enterococcus faecalis* culture, previous complication of surgery or medical care, previous valvular disease, hemoglobin, previous Charlson Score, previous Elixhauser score, hospitalization in the previous 180 days, hospitalization in the previous 30 days, marital status, pneumococcal vaccination in the previous 10 years, pneumococcal vaccination in the previous 1 year, previous skin/ subcutaneous infection, previous urinary tract infection, race, and treating specialty. Excluded one patient with a missing creatinine and one patient with a missing hemoglobin.

### Antimicrobial Utilization

**Fig 3:**
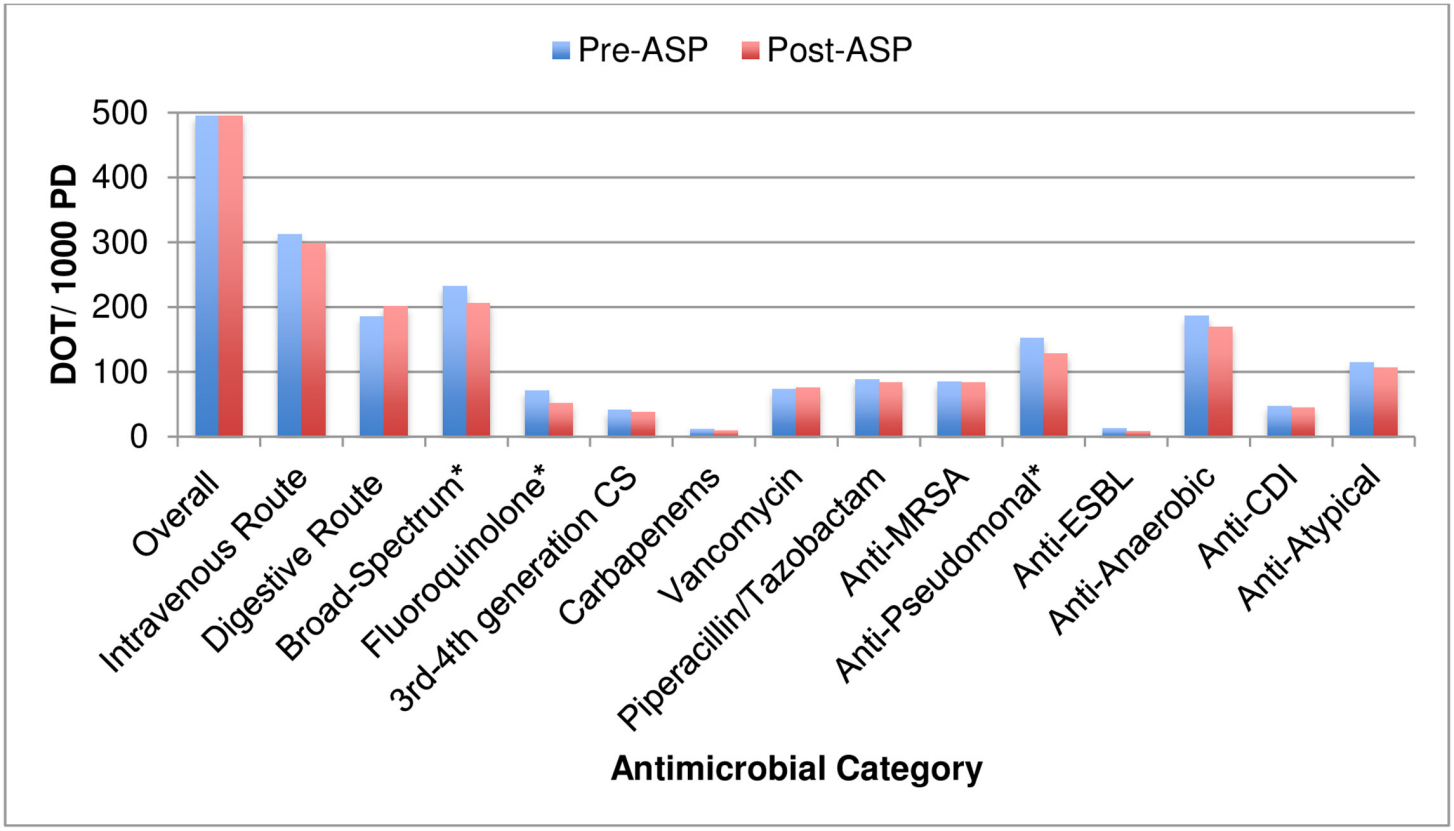
Antimicrobial Use Comparison Pre- and Post- Antimicrobial Stewardship Program (ASP) Implementation. CS = cephalosporins; CDI = *Clostridium difficile* infection; ESBL = extended spectrum β-lactamase, MRSA = methicillin-resistant *Staphylococcus aureus*. * = p<0.05.

There was no difference in the overall mean DOT/1000PD between the pre- and post-ASP periods (Table 4). However, there was a significant (p<0.05) decrease in mean broad-spectrum use (-11.3%), specifically driven by fluoroquinolones (-27.0%) and anti-pseudomonals (-15.6%). IV use decreased (-4.6%, p = 0.43) and digestive use increased (+8.3%, p = 0.26). All other antimicrobial categories assessed decreased non-significantly, except vancomycin (Fig 3).

**Table 4:** Mean Monthly Antimicrobial Use in Days of Therapy per 1000 Patient Days (DOT/1000PD) by Period.

Antimicrobial Category	Pre-Antimicrobial Stewardship Period (DOT/1000 PD)	Post-Antimicrobial Stewardship Period (DOT/1000 PD)	Percent Change in Antimicrobial Use (%)
Overall	494.7 ± 54.1	494.9 ± 70.4	0.0
Intravenous Route	312.3 ± 38.9	298.0 ± 48.4	-4.6
Digestive Route^a^	185.3 ± 26.1	200.6 ± 37.2	+8.3
Broad-Spectrum*^b^	231.9 ± 29.3	205.6 ± 29.0	-11.3
Fluoroquinolone*	71.0 ± 8.6	51.8 ± 11.1	-27.0
3^rd^-4^th^ Generation CS	40.9 ± 12.8	37.6 ± 7.4	-8.1
Carbapenems	11.3 ± 7.7	8.7 ± 4.3	-23.0
Vancomycin	73.3 ± 12.8	75.8 ± 20.3	+3.4
Piperacillin/Tazobactam	88.1 ± 10.9	83.7 ± 18.9	-5.0
Anti-MRSA^c^	83.9 ± 15.2	82.7 ± 25.0	-1.4
Anti-Pseudomonal*^d^	152.1 ± 18.8	128.3 ± 22.1	-15.6
Anti-ESBL^e^	12.3 ± 7.3	8.2 ± 4.3	-33.3
Anti-Anaerobic^d^	186.1 ± 26.9	168.7 ± 28.5	-9.3
Anti-CDI^g^	46.9 ± 18.9	44.5 ± 16.2	-5.1
Anti-Atypical^h^	114.0 ± 25.5	105.7 ± 18.3	-7.3

Data are mean ± standard deviation or % change. The DOT represents the sum of the days for which a single antimicrobial was administered, regardless of the number of doses administered or dosage strength.

CS = cephalosporins; CDI = *Clostridium difficile* infection; ESBL = extended spectrum β-lactamase, IV = intravenous; MRSA = methicillin-resistant *Staphylococcus aureus*; PO = oral; PR = rectal.

* = p<0.0

^a^ = Digestive route use included oral and rectal antimicrobials.

^b^ = Broad-spectrum antimicrobial use included β-lactams/ β-lactamase inhibitors, 3^rd^ and 4^th^ generation cephalosporins, carbapenems, and fluoroquinolones.

^c^ = Antimicrobials with activity against MRSA, included tigecycline, daptomycin, telavancin, vancomycin IV, quinupristin/dalfopristin, linezolid, and ceftaroline.

^d^ = Antimicrobials with activity against *Pseudomonas aeruginosa*, included ticarcillin/clavulanate, piperacillin/tazobactam, ceftazidime, cefepime, imipenem, meropenem, doripenem, amikacin, gentamicin, tobramycin, ciprofloxacin, levofloxacin, polymyxin B, colistin, and fosfomycin.

^e^ = Antimicrobials with activity against ESBLs, included tigecycline, carbapenems, polymyxin B, colistin, and fosfomycin.

^f^ = Antimicrobials with activity against anaerobes, included tigecycline, β-lactams/ β-lactamase inhibitors, cefoxitin, cefotetan, carbapenems, clindamycin, moxifloxacin, and metronidazole.

^g^ = Antimicrobials with activity against *Clostridium difficile*, included vancomycin PO/PR, fidaxomicin, and metronidazole PO.

^h^ = Antimicrobials with activity against atypical pneumonia pathogens, included tetracyclines, tigecycline, macrolides, and fluoroquinolones.

ITS analyses demonstrated several significant level changes for antimicrobial use, including digestive, anti-CDI, and anti-anaerobic use (Table 5). The only significant change in month-to-month trend observed was with carbapenems (+1.5 DOT/1000PD per month; 95% CI 0.1–3.0, p = 0.035).

**Table 5:** Significant Changes in Antimicrobial Use using Interrupted Time Series Analysis.

Antimicrobial Category	DOT/1000PD per month	95% Confidence Interval	P-value
**Change in level**			
Digestive Route^a^	+110.1	15.2–205.0	0.025
Anti-Anaerobic^b^	+110.6	43.2–177.1	0.003
Anti-CDI^c^	+76.1	32.0–120.1,	0.002
**Change in trend**			
Carbapenems	+1.5	0.1–3.0	0.035

Models for change in level contained only the baseline trend and level change.

Models for change in trend contained the baseline trend, level change, and intervention trend.

CDI = *Clostridium difficile* infection; DOT/1000PD = Days of therapy per 1000 patient days; PO = oral; PR = rectal

^a^ = Digestive route use included oral and rectal antimicrobials.

^b^ = Antimicrobials with activity against anaerobes, included tigecycline, β-lactams/ β-lactamase inhibitors, cefoxitin, cefotetan, carbapenems, clindamycin, moxifloxacin, and metronidazole.

^c^ = Antimicrobials with activity against *Clostridium difficile*, included vancomycin PO/PR, fidaxomicin, and metronidazole PO.

### Antimicrobial Costs

Total antimicrobial costs decreased 14% pre- to post-ASP, with a non-significant 5.3% decrease in mean antimicrobial costs/1000PD (p = 0.5). The cost for fluoroquinolones decreased 29% pre- to post-ASP (p<0.05). IV (-4.2%), digestive (-7.6%), and broad-spectrum (-9.5%) costs all decreased non-significantly.

ITS demonstrated several significant increases in antimicrobial costs immediately following the implementation of ASP. While the level of anti-CDI, anti-anaerobic, and broad-spectrum costs increased, this increase was not sustained during the post-ASP period.

### Antimicrobial Resistance

No significant changes in antimicrobial resistance were observed for any of the Gram-positive or Gram-negative organism-antimicrobial combinations assessed (Table 6), except for *Klebsiella pneumoniae*, in which several significant (p<0.05) increases in resistance were observed.

**Table 6:** Antimicrobial Resistance in Pre- and Post-Antimicrobial Stewardship Periods.

Organism	Antimicrobial Tested	Pre-Antimicrobial Stewardship Period, Percent Resistance (n isolates tested)	Post-Antimicrobial Stewardship Period, Percent Resistance (n isolates tested)
**Gram-Positive Organisms**			
*Enterococcus faecalis*	Ampicillin	0 (114)	0 (124)
	Gentamicin	28.4 (102)	26.6 (124)
	Vancomycin	6.4 (109)	3.2 (125)
*Enterococcus faecium*	Ampicillin	90.0 (20)	77.8 (18)
	Gentamicin	0 (16)	5.3 (19)
	Tetracycline	100 (13)	90.9 (11)
	Vancomycin	89.5 (19)	61.1 (18)
MRSA	Clindamycin	43.0 (121)	44.1 (143)
	Gentamicin	0.7 (146)	0 (162)
	Tetracycline	2.7 (146)	3.7 (162)
	Trimethoprim-sulfamethoxazole	0 (146)	1.2 (162)
	Vancomycin	0.7 (146)	0 (161)
MSSA	Clindamycin	20.0 (168)	27.0 (163)
	Erythromycin	32.7 (168)	36.2 (163)
	Gentamicin	2.1 (190)	1.1 (179)
	Penicillin	81.1 (190)	77.1 (179)
	Tetracycline	3.2 (190)	2.2 (179)
	Trimethoprim-sulfamethoxazole	2.1 (190)	1.1 (179)
	Vancomycin	0 (190)	1.1 (179)
**Gram-negative Organisms**			
*Klebsiella pneumoniae*	Amikacin	2.3 (128)	4.0 (124)
	Ampicillin	96.9 (128)	100 (124)
	Ampicillin-sulbactam	20.3 (128)	26.6 (124)
	Aztreonam	9.4 (128)	13.7 (124)
	Cefazolin*	11.7 (128)	23.4 (124)
	Cefepime*	4.7 (128)	12.1 (124)
	Ceftriaxone*	6.3 (128)	13.7 (124)
	Ciprofloxacin*	10.3 (126)	20.7 (121)
	Gentamicin*	6.3 (128)	15.3 (124)
	Imipenem	0 (127)	0 (122)
	Piperacillin-tazobactam*	0 (117)	5.6 (107)
	Trimethoprim-sulfamethoxazole*	9.4 (128)	21.0 (124)
*Acinetobacter baumannii*	Amikacin	12.5 (8)	9.1 (11)
	Cefepime	25.0 (8)	18.2 (11)
	Ceftazidime	37.5 (8)	9.1 (11)
	Ciprofloxacin	12.5 (8)	9.1 (11)
	Gentamicin	12.5 (8)	18.2 (11)
	Imipenem	14.3 (7)	20.0 (10)
	Trimethoprim-sulfamethoxazole	12.5 (8)	18.2 (11)
*Pseudomonas aeruginosa*	Amikacin	13.1 (130)	12.4 (121)
	Aztreonam	26.9 (130)	31.4 (121)
	Cefepime	7.6 (131)	10.7 (122)
	Ceftazidime	12.2 (131)	10.0 (120)
	Ciprofloxacin	22.7 (132)	20.7 (121)
	Gentamicin	19.7 (132)	23.8 (122)
	Imipenem	11.4 (132)	10.7 (122)
	Piperacillin-tazobactam	3.9 (128)	4.4 (114)
	Tobramycin	0.8 (131)	5.8 (121)
*Escherichia coli*	Amikacin	0 (243)	1.0 (256)
	Ampicillin	44.0 (243)	42.0 (257)
	Ampicillin-sulbactam	39.9 (243)	35.9 (256)
	Aztreonam	6.2 (243)	5.1 (257)
	Cefazolin	14.0 (243)	14.0 (256)
	Cefepime	4.1 (243)	3.9 (257)
	Ceftriaxone	4.9 (243)	5.1 (256)
	Ciprofloxacin	25.9 (243)	20.1 (254)
	Gentamicin	22.0 (243)	18.0 (257)
	Imipenem	0 (243)	0 (257)
	Piperacillin-tazobactam	2.1 (234)	2.1 (243)
	Trimethoprim-sulfamethoxazole	23.0 (243)	18.0 (255)

MRSA = methicillin-resistant *Staphylococcus aureus*; MSSA = methicillin-sensitive *Staphylococcus aureus*.

* = p<0.05.

### Patient Safety

The mean rate of CDI/10,000PD was 14.2 ± 10.4 pre-ASP and 13.8 ± 10.0 post-ASP (p = 0.94). No significant changes in level or trend of CDI/10,000PD per month were observed. Fewer patients experienced ADEs post-ASP (6.0% vs. 4.4%, p = 0.06).

## Discussion

Currently, there is no consensus on which metrics are the most optimal to adequately assess the impact of an ASP.[29] Our study provides a detailed assessment of the impact of an ASP on clinical outcomes, antimicrobial utilization, costs, resistance, patient safety, and process metrics. Due to the challenges associated with outcomes assessment, most studies to date have focused on measuring the impact of an ASP on just one or two metrics, most commonly antimicrobial utilization and costs.

While median LOS was 1 day shorter post-ASP, this difference was not statistically significant in propensity matched or adjusted analyses. Despite patients being generally sicker post-ASP (higher Charlson and Elixhauser scores and higher prevalence of several comorbidities), ASP interventions may have led to improved quality of care, enabling patients to be discharged sooner. Nonetheless, in general, ASP implementation had a limited impact on the clinical outcomes assessed. These findings are similar to most studies, which have demonstrated little to no impact of prospective audit and feedback ASPs on clinical outcomes, including LOS, [8–17] mortality, [8–10, 12–17] and 30-day readmission.[10, 14] This may be because, a large number of factors affect clinical response and outcomes, and therefore the independent effect of ASP interventions on these outcomes may be negligible.[29] Additionally, while in adjusted analyses 30-day mortality was higher post-ASP, this included deaths due to all-causes. The Centers for Medicare and Medicaid Services (CMS) 30-day risk standardized mortality rates for congestive heart failure at the Providence VA Medical Center were higher during the post-ASP period than the pre-ASP period.[30]. Also, antimicrobial stewardship interventions are likely to have a greater impact on 7- and 14-day mortality and inpatient mortality, which did not differ between periods.

We also measured the effect of our ASP on antimicrobial resistance. In another study, reduction of broad-spectrum antimicrobial use was not associated with improvements in the hospital antibiogram.[31] As with clinical outcomes, the factors associated with antimicrobial resistance are complex and involve many factors such as infection control, antimicrobial use within and outside the hospital, and patient colonization and immune status. Therefore, it can be challenging for an ASP to demonstrate a favorable impact on antimicrobial resistance.[32] Moreover, it can take years before a program has an effect on antimicrobial resistance.

In our assessment of antimicrobial use, we did not observe a decrease in overall mean antimicrobial use, which may be related to the appropriateness of antimicrobial utilization prior to implementation of our ASP. It is estimated that 50% of antimicrobial use in hospitals is inappropriate.[7] However, in our study, only 37% of patient records reviewed were deemed to require intervention. Since 2004, a clinical pharmacist with formal training in infectious diseases has provided the PVAMC expert consultation, an antimicrobial guide with empiric treatment recommendations and an antibiogram, and educational programs. Additionally, several broad-spectrum antimicrobials have been restricted since before the implementation of our ASP. Therefore, at baseline appropriate antimicrobial use at the PVAMC may have been relatively high.

Though overall use did not decrease, we did see significant reductions in broad-spectrum, fluoroquinolone, and anti-pseudomonal use post-ASP. Our ASP improved the use of these broad-spectrum antimicrobials, through appropriate antimicrobial de-escalation and optimization. We also observed a reduction in mean carbapenem use post-ASP, however ITS demonstrated an increasing trend in carbapenem use. This highlights the importance of conducting ITS analysis to uncover immediate and sustained changes in outcome measures over time. This increasing trend in carbapenem use may be due in part to rotating medical residents. At the PVAMC, residents are the primary antimicrobial prescribers, and they rotate out of the PVAMC to other local hospitals every month. At the time of this study, the PVAMC had the only comprehensive multidisciplinary ASP in the area. Moreover, there was no formal ASP at the flagship hospital that the residents rotate through. Therefore residents may have not been used to the ASP service. In a recent study, investigators demonstrated an improvement in the level of audited antimicrobials but no change in the trend, which was also likely due to residents changing to different departments or institutions frequently.[15] Monthly introductions of the house-staff and new medical residents to our ASP and other educational material such as newsletters or posters, may increase residents’ awareness and connection to our service, and improve the ASP culture at the PVAMC. The increasing trend in carbapenem use may also be related, in part, to the significant increases in resistance observed for *Klebsiella pneumoniae*.

Measuring the impact of ASPs on patient safety is also important. Rates of CDI were similar pre- and post- ASP. This is not surprising, as CDI rates were already low prior to ASP implementation, likely due to strong infection control practices. Infection control has had guidelines for the prevention and control of CDI since before the pre-ASP period. Guidelines include barrier methods, contact precautions, hand hygiene, and environmental infection control methods. Additionally, we observed a trend towards decreased ADEs post-ASP. Due to the difficulties in obtaining accurate data, very few studies have assessed the impact of ASPs on ADEs.[33]

Our ASP did not have a significant impact on the clinical outcome measures assessed. This may be due in part to the outcomes metrics chosen. As previously mentioned, the most optimal metrics to demonstrate the value of an ASP are largely unknown.[29] As we continue to strengthen our program, we look to assess additional metrics such as infection-related clinical outcomes and total costs of care, not just drug costs, and to assess the impact of these outcomes over a longer follow-up period. Additionally, almost 90% of our recommendations were made through written notes. Our feedback may have had a greater impact if it was provided through face-to-face communication or phone calls directly to the provider. Notes left in the chart are unlikely to be seen in a timely manner.[34] Busy providers may miss or ignore notes. Moreover, the impact of our program may be limited by the timeliness of final culture results. At the PVAMC, traditional microbiologic testing (culture and susceptibility) is primarily utilized, which is suboptimal in providing rapid organism identification and susceptibility results.[35] Previous research by our group has demonstrated that the median time to final culture results ranged from 3–5 days at our facility and regionally. Therefore, incorporation of rapid diagnostic testing (RDT) could significantly enhance the impact of our ASP.[35] RDT has the potential to improve clinical outcomes, costs, and resistance rates by decreasing the time to appropriate therapy and quickly stopping unnecessary therapy.

There are several limitations to our study. The quasi-experimental design is associated with a number of inherent limitations, including the potential for confounding bias. However, we did our best to control for differences between patients in the pre- and post-ASP periods through propensity score adjustment and matching. Still, differences in unmeasured factors may exist between the groups. We may not have been able to capture all residual confounding, and having a generally sicker population in the post-period may bias estimates of differences in clinical outcomes towards the null. Of note, while there were no outbreaks at the PVAMC in either period, the 2012–2013 influenza season started earlier in Rhode Island and was more severe than previous years (including the 2010–2011 season).[36, 37] Significantly more patients in the post-ASP period had a diagnosis of influenza than in the pre-ASP period.

As with any study that utilizes secondary data sources, this study may be limited by the accuracy of the data contained within the various data sources. While we attempted to develop accurate definitions for outcomes and potential confounders, misclassification bias may still affect our results. However, the VA has used an electronic medical record for over 15 years, from which the VA research databases are extracted, and the accuracy and completeness of several VA datasets has been verified in previous studies.[38–41]

It is unclear how long it takes for changes in antimicrobial utilization to subsequently impact resistance rates and clinical outcomes. Our study only assessed the first year post-implementation. Therefore, it is possible that we did not allow enough time to observe an effect, as it may take several years of follow-up. However, we utilized interrupted time series analysis which is the strongest approach to quantify the effects of an intervention over time for quasi-experimental studies.[27] Additionally, since few deaths occurred, we may not have been able to detect a difference between groups. Finally, we conducted a single center VA study and the generalizability of our study may be limited to the VA setting. VA patients tend to differ from the general population in terms of patient demographics and comorbidities, and the VA has unique resources, which may assist with ASP efforts. Nonetheless, our study could serve as an example to other burgeoning stewardship programs that are interested in analyzing the potential effectiveness of their interventions.

## Conclusions

Our prospective audit and feedback program was associated with improvements in broad-spectrum antimicrobial use. While median LOS was shorter post-ASP, clinical outcomes were similar pre- and post-ASP. Resistance, costs and patient safety indicators did not significantly change, but these changes may have a positive impact long term. Further measures, such as increased use of RDT, increased direct verbal feedback, and additional outcomes metrics, may be necessary moving forward. Moreover, as our ASP has now been in effect for over three years, we look to continue to measure the sustained impact of our program over time.

Overall, prospective audit and feedback has the potential to improve antimicrobial use and outcomes, and contain bacterial resistance. Our program demonstrated a trend towards decreased length of stay, broad-spectrum antimicrobial use, antimicrobial costs, and adverse drug events. While these results were not statistically significant, we believe that these findings have important clinical impact to the care of our patients.

## Supporting Information

S1 File (CSV)
